# Atypical Temporal Dynamics of Resting State Shapes Stimulus-Evoked Activity in Depression—An EEG Study on Rest–Stimulus Interaction

**DOI:** 10.3389/fpsyt.2019.00719

**Published:** 2019-10-15

**Authors:** Annemnarie Wolff, Sara de la Salle, Alana Sorgini, Emma Lynn, Pierre Blier, Verner Knott, Georg Northoff

**Affiliations:** ^1^University of Ottawa Institute of Mental Health Research, Ottawa, ON, Canada; ^2^Department of Cellular and Molecular Medicine and Neuroscience, Faculty of Medicine, University of Ottawa, Ottawa, ON, Canada; ^3^Brain and Mind Research Institute, University of Ottawa, Ottawa, ON, Canada; ^4^Department of Psychology, Faculty of Social Sciences, University of Ottawa, Ottawa, ON, Canada

**Keywords:** EEG, Depression, resting state, Peak frequency, alpha oscillations, theta oscillations

## Abstract

Major depressive disorder (MDD) is a complex psychiatric disorder characterized by changes in both resting state and stimulus-evoked activity. Whether resting state changes are carried over to stimulus-evoked activity, however, is unclear. We conducted a combined rest (3 min) and task (three-stimulus auditory oddball paradigm) EEG study in *n*=28 acute depressed MDD patients, comparing them with *n*=25 healthy participants. Our focus was on the temporal dynamics of both resting state and stimulus-evoked activity for which reason we measured peak frequency (PF), coefficient of variation (CV), Lempel-Ziv complexity (LZC), and trial-to-trial variability (TTV). Our main findings are: i) atypical temporal dynamics in resting state, specifically in the alpha and theta bands as measured by peak frequency (PF), coefficient of variation (CV) and power; ii) decreased reactivity to external deviant stimuli as measured by decreased changes in stimulus-evoked variance and complexity—TTV, LZC, and power and frequency sliding (FS and PS); iii) correlation of stimulus related measures (TTV, LZC, PS, and FS) with resting state measures. Together, our findings show that resting state dynamics alone are atypical in MDD and, even more important, strongly shapes the dynamics of subsequent stimulus-evoked activity. We thus conclude that MDD can be characterized by an atypical temporal dynamic of its rest–stimulus interaction; that, in turn, makes it difficult for depressed patients to react to relevant stimuli such as the deviant tone in our paradigm.

## Introduction

### Temporo-Spatial Dynamics Shape Rest and Task States

Major depressive disorder (MDD) is a complex psychiatric disorder that includes affective, cognitive, vegetative, sensorimotor, social, and perceptual changes (1). Neuronally, changes in stimulus-evoked activity in response to especially affective and cognitive stimuli have been reported in MDD, including event-related potential (ERP) changes during auditory target detection paradigms as well as atypical power in theta (5–8 Hz) and/or alpha (7–13 Hz) frequency ranges (2–6). The origin of these neuronal changes in stimulus-evoked activity in MDD, however, remains unclear. 

Recent studies have demonstrated changes in the resting state in MDD (7). FMRI resting state studies show atypical functional connectivity, especially in anterior regions of the prefrontal cortex, as well as in other networks such as the default-mode network (8), while EEG resting state studies demonstrate atypical activity, especially in theta and alpha frequencies in MDD (7, 9, 10). Given the overlap of both rest and task findings in theta and alpha frequencies, one would suggest that resting state changes may also shape stimulus-evoked activity in depression. This link is yet to be shown. 

The brain’s resting state, or spontaneous activity, can be characterized by an intrinsic temporo-spatial dynamic (11–13). Such dynamics concern, among other features, various frequency bands, such as theta and alpha (14). Importantly, the strength of these frequencies can be characterized by peak frequencies (15–17) which, for instance, has been shown to be atypically low in the resting state of first-episode psychosis patients (17). To our knowledge, no studies have examined peak frequency in MDD specifically; this temporo-spatial dynamic measure is yet to be probed in MDD. Other resting state studies, however, have found greater functional connectivity in the theta and alpha bands in MDD when compared with healthy controls (18) as well as a decrease in long-range temporal correlations (19). A recent review found that studies of alpha asymmetry in MDD patients had inconsistent findings (20), despite its early promise (21).

The brain’s temporo-spatial dynamic extends beyond its spontaneous activity to its stimulus-evoked activity. Two studies have demonstrated the interaction of prestimulus activity levels with poststimulus activity (22–24) by dynamic measures which account for complexity and variance. 

One recent EEG study in healthy participants showed the relationship of prestimulus complexity, as measured with Lempel-Ziv Complexity (LZC) (25), with poststimulus trial-to-trial variability (TTV) (26–29). LZC, a measure from information theory (30), quantifies patterns, and their repetition, within a sequence, with higher values of complexity indicating less structure and predictability in the sequence (25). This study (29) showed that prestimulus complexity related to poststimulus variability reduction measured through TTV, and that the poststimulus decrease in LZC and variability may capture the increase in regularity of neural activity after stimulus onset, which has been shown in computational models (31). Whether LZC and TTV, or their relation, are atypical in MDD, however, is yet to be studied. 

A measure that has been applied recently is the change in peak frequency (PF) in specific bands, the alpha and theta bands in particular (17, 32, 33). The PF is defined as the frequency within a specified band—alpha, theta, and beta—which has the maximal power (for example, the peak frequency in the alpha band could change from 9 to 10 Hz when doing a task (16)) (32–34).

The PF and its change can be measured during the resting state or in response to a stimulus—PF can increase in alpha when performing a task (16), termed frequency sliding (FS) (32). Changes in PF is based on the neuronal principle that the firing rate of a neuron is proportional to the strength of its input (35); it has been shown (32) in both computational models and human EEG rest and task data that if input to a neural network increases, the “speed” of oscillations of a specific frequency band, and thus its PF, will increase accordingly. As a result, FS has shown to be an index, which can be measured using scalp EEG, of input to a neural network (32).

### General and Specific Aims

The general aim of our combined rest and task EEG study in MDD consisted in investigating how changes in the resting state’s temporo-spatial dynamics shape stimulus-evoked activity. Our study can thus be conceived of as an example of rest–stimulus interaction (23, 24, 36) in MDD. For that purpose, we investigated resting state and task activity (auditory oddball) in MDD. 

We first applied the above-mentioned dynamic measures—PF and its variance, and power—to the resting state in MDD and a control group. Based on previous findings (16, 17), we hypothesized lower PF in the theta (4–8 Hz) and alpha (7–13 Hz) bands in the MDD group in comparison to the control group.

Next, we applied the dynamic measures of LZC and TTV to stimulus-evoked activity in MDD and the control group. Given the previous findings of atypical ERP, we hypothesized decreases in both poststimulus TTV change and LZC poststimulus change in MDD. The employment of an auditory oddball paradigm also allowed us to distinguish between attention-demanding task-relevant deviant tones and task-irrelevant standard tones. Consistent with previous findings in MDD (2, 4, 6), we hypothesized atypical LZC and TTV results in the deviant tones in particular.

After the LZC and TTV analysis, we measured the dynamic FS—and corresponding power sliding (PS)—to the same deviant and standard tones in the theta and alpha frequency bands. We hypothesized that these measures in the MDD group would differ significantly from the healthy controls in the deviant tones, but not the standards. 

Finally, we sought to relate rest and task measures. We hypothesized that the dynamical measures of the resting state (PF, power) correlate with poststimulus TTV and LZC change in both groups. The temporo-spatial dynamics of the resting state, including its changes, may thus be conveyed to the subsequent stimulus-evoked activity and its own dynamics.

## Methods

### Participants

Twenty-eight participants with Major Depressive Disorder (MDD) (age: mean = 54±18 years, range = 16–73 years; 19 female) and twenty-five healthy controls (age: mean = 46±16 years, range = 21–70 years; 14 female) completed this study. MDD participants were recruited from an outpatient population with major depressive disorder (MDD) that were participating in a larger adjacent clinical trial. Clinical assessments and diagnosis of depression were conducted with a study psychiatrist. MDD participants were evaluated with the Montgomery-Åsberg Depression Rating Scale (MADRS) (37) and found to have scores between 14 and 37 (mean = 28±6) (inclusive) at the time of the electroencephalography (EEG) session. 

Exclusion criteria for the MDD participants were the following: DSM-IV disorder other than MDD; depression secondary to severe medical illness; positive urine drug screen for illicit substances or non-prescribed medicine; substance or alcohol abuse or dependence 6 months prior to enrollment; diagnosis of a neurological disorder; pregnancy or lactation; an unstable clinical finding that might be negatively affected by the treatment of the larger clinical trial; liver function tests three times the upper normal limit; inadequately corrected hypothyroidism or hyperthyroidism; clinically significant deviation from the reference range in clinical laboratory test results; imminent suicidal or homicidal risk; and participation in another clinical treatment study with a drug that had not yet received approval. 

Age- and sex-matched healthy adults were recruited from the local community as a control group. Participants were assessed using the SCID non-patient version (SCID-NP; 38) and the Family Interview for Genetic Studies (FIGS; 39), following an initial telephone session and a subsequent face-to-face interview.

The Beck Depression Inventory (BDI) score was used to verify that the control participants did not have depression. We administered the BDI to the patient group as part of the larger adjacent clinical trial; BDI scores before and after treatment in the clinical study were compared. Only the BDI scores recorded before the treatment initiation were used here. MDD Participants had BDI scores greater than 13 (mean = 28±10), while all healthy control participants had BDI scores below 13 (mean = 1±2).

The experimental protocols were approved by the research ethics committee of the University of Ottawa Institute of Mental Health Research, and the study was carried out with their permission. Written informed consent was obtained from each participant prior to study participation.

### EEG Session

EEG data were recorded using a Brain Vision EasyCap with 32 Ag/AgCl electrodes at a sampling rate of 500 Hz. Electrode AF_z_ served as the ground and an additional nose electrode served as the reference during recording.

Additional channels were added for independent component analysis (ICA) decomposition: vertical ocular (above and below the left eye), and horizontal ocular (the outer canthi of the right and left eyes). The impedance of all channels was maintained at less than 5 kΩ during recording. 

Prior to beginning the task, participants completed three-minute resting state EEG recordings with their eyes closed.

The task was an auditory oddball paradigm (40) and was presented to participants using the Presentation software (Neurobehavioral Systems, Albany, CA, USA). Participants were presented with 800 tones in four blocks (200 tones per block). Eighty percent of the tones, standard tones, were 1,000 Hz and 70 dB pure tones 336 ms in length. Ten percent were deviant tones at 700 Hz (70 dB pure tones lasting for 336 ms, identical to standards). Participants were instructed to respond to the deviant tones by a button press. The remaining 10% were novel non-target—participants did not respond by a button press—environmental sounds (ie., dog bark, horn, etc) at 65–75 dB for 169–399 ms. Between these stimuli, participants were presented with a fixation cross for 1 s. Reaction times to target responses were recorded.

The standard and deviant stimuli were the focus of this study as they were alike; both were pure tones with the same length and power. The novel stimulus had varying lengths and was not a pure tone, so not comparable to the other two stimuli. The novel stimuli, however, were analyzed with TTV and LZC for differences between MDD and controls. This was done only to determine if any differences found in the deviant stimuli were due to the infrequent presentation of the stimulus.

### EEG Preprocessing

All EEG data preprocessing was completed using EEGLAB (v14) (41), which required MATLAB (The MathWorks) v2018b, including the use of the Optimization, Statistics and Signal Processing Toolboxes. All statistical analyses were completed in MATLAB v2018b.

The raw continuous data was low- and high-pass FIR filtered from 1Hz to 40Hz, and then visually inspected. If channels were flat longer than 5 s, had less than 0.80 correlation with neighboring channels, or had line noise greater than four standard deviations difference compared to other channels, they were spherically interpolated.

The task data was then epoched—with no baseline correction—to stimulus onset. All files were re-referenced to the surface Laplacian reference, according to the methods of previous studies (42, 43), to spatially filter the data; activity recorded at the specified electrode had a higher correspondence with cortical activity just below it than had an alternative reference montage been used. 

All stationary artifacts, specifically eye movements, were reduced using Independent Component Analysis (ICA) and the Multiple Artifact Rejection Algorithm (44, 45).

### Resting State Measures: Peak Frequency (PF), Coefficient of Variation (CV), Power

We began our analysis by investigating the resting state data. All preprocessed resting state files were first cut to the same length (85,000 data points). According to previous studies (16, 17, 29, 32, 34, 46, 47), our *a priori* interests were focused on activity in the alpha (7–13Hz) and theta (4–8Hz) bands. For this reason, and due to the findings of dmPFC in rest-task interactions (36) and its implication in MDD (48–50), all analyses were done with electrodes Fz for theta (51, 52) and broadband activity, and Pz for alpha related activity (53, 54). All data analysis was completed in MATLAB (v2018b).

The peak frequency (PF) was calculated according to the methods of MX Cohen (32). Specifically, the preprocessed broadband data was first FIR bandpass filtered—with 15% transition zones added to each edge of the filtered range according to the published methods (32). The data were then Hilbert transformed to get the analytic signal, and the phase-angle timeseries was extracted using MATLAB’s *angle* function. To compute the frequency of the peak within the filtered band, the first derivative of the phase-angle timeseries was taken. Finally, a median filter (filter order 10) was applied to the timeseries according to the published methods of this approach (32). The mean over the full three-minute resting state of the peak frequency was calculated at electrode Fz for the theta band (4–8 Hz) and Pz for the alpha band (8–13 Hz), as was the power in the same electrodes for the same bands.

The coefficient of variation (CV) has previously been measured in neuroscientific data on single neuron spike trains, as well as data from larger arrays (55, 56). Recently, however, it has also been applied to EEG data (28, 57). The CV, also termed the Fano Factor (58), is a measure of relative variability as 

(1) $$CV=\frac{\sigma }{\mu }$$

with σ being the standard deviation of the peak frequency in the resting state and µ being the mean. We measured the CV as it quantifies the stability of the peak and normalizes the standard deviation as it divides by the mean.

### Trial-To-Trial Variability

Trial-to-Trial Variability (TTV) was calculated according to the methods of previous studies (23, 27–29). TTV is defined as the variability changes over trials with respect to variability at stimulus onset:

(2) $$TTV(t)=\frac{\sigma_{ot}(t)-\sigma_{ot}(0)}{\sigma_{ot}(0)}x100$$

where σ*_ot_* (*t*) is the SD of the EEG signal over trials at timepoint *t* and σ*_ot_* (0) is the SD over trials at stimulus onset. Therefore, each poststimulus timepoint is relative to stimulus onset.

In the task, eighty stimuli were deviants with many more standards. To compare the two stimuli, in this and subsequent analyses, eighty randomly chosen trials of standard stimuli were extracted. Therefore, all measures contained the same number of deviant and standard trials, with the standards randomly chosen.

To compare the TTV between stimuli and groups, the area under the curve (AUC) between stimulus onset (0 ms) and 500 ms was computed and compared statistically.

### Lempel-Ziv Complexity Analysis

As computed in a previous study (29), we applied a measure of complexity from information theory (30) to the non-baseline corrected data. Lempel-Ziv Complexity (LZC) was calculated from previous studies (25, 29, 59) in MATLAB v2018b using a custom script. In both the pre- and post-stimulus periods for which LZC was calculated, 300 ms of the signal was measured.

The EEG signal was first converted into a binary sequence with each data point in a timeseries *x*(*i*) being converted to a symbol in the sequence *s*(*i*):

(3) $$s(i)=\;\{\begin{array}{c} 0\;\;\;if\;x(i)<T_{d}\\ 1\;\;\;if\;x(i)\geq \;T_{d} \end{array}$$

where *T* *_d_* is the threshold (25), the median of the timeseries here. The median was chosen according to previous studies (25, 29) as it is unaffected by extreme values in a sequence. This sequence *s*(*i*) is then scanned from left to right. The complexity measure *c*(*n*) is increased by one each time a new sequence of consecutive values occurs (25). Finally, the complexity *C*(*n*) is normalized to control for signal length:

(4) $$C(n)=\frac{c(n)}{\frac{n}{\log_{2}(n)}}$$

where *n* is the length of the sequence. LZC has no units.

The difference between the pre- and post-stimulus was calculated (poststim LZC minus prestim LZC) and this was compared between MDD and controls (CON).

### Peak Frequency Sliding (FS) and Power Sliding (PS) During Task

Calculated in the same way as the PF in the resting state (see above) according to the methods of MX Cohen (32), the peak frequency sliding (FS) in theta (4–8 Hz) and alpha (7–13 Hz) was calculated. For each of the deviant and standard trials, the FS was computed, and the mean over all the trials was then calculated. To account for any changes in PF at stimulus onset between groups (see **Supplementary Figure 1**), the data were converted to percent change; the PF in the task is the percent change relative to stimulus onset.

Next, to calculate the power sliding (PS), the same analysis was done, with one difference: rather than extract the phase-angle timeseries, the modulus (absolute value) of the Hilbert transform was extracted. This was then squared—at each timepoint—to give a timeseries of the power in the specified band. As with the FS, the PS was converted to percent change relative to stimulus onset to correct for any differences in power between MDD and controls.

Finally, the AUC in 100 ms intervals was calculated for both measures and compared statistically. The time intervals in which the AUC was measured were as follows: 376–476 ms for the alpha FS; 150–250 ms for the alpha PS; 400–500 ms for the theta FS; 166–266 ms for the theta PS.

### Task-Task Correlation: Trial-To-Trial Variability (TTV) and Frequency/Power Sliding (FS/PS)

To compare the results from the TTV analysis and the FS and PS analysis, two-tailed Spearman correlations were performed for both stimuli on all participants (MDD and CON) together. The significance level for all correlations was 0.05. To correct for multiple statistical tests, the Benjamini-Hochberg False Discovery Rate (60) was applied to all *p-*values. The *p*-values stated in the results and figures are therefore corrected for multiple comparisons.

### Rest-Task Correlation: Resting State PF/CV and Task (TTV, LZC, FS, and PS)

Finally, to link the resting state findings (PF, CV, and Power) to those from the task (TTV, LZC, FS, and PS), two-tailed Spearman correlations were performed on all participants (MDD and CON) together. Again, the significance level for all correlations was 0.05. As above, the Benjamini-Hochberg False Discovery Rate (60) was applied to all *p*-values, so all *p*-values reported in the results and figures have been corrected for multiple comparisons.

### Statistical Analyses

All statistical analyses were done in MATLAB v2018b, using the Statistics toolbox, at a significance level of 0.05.

It has been shown that large interindividual differences between participants exists and, in fact, is a particular focus of recent research (29, 44, 61–65). Typical parametric tests, however, often fail to account for a factor that has an effect in many individuals since they focus on the average effect (66). With that in mind, and at the direction of a study on that exact idea (66), to compare the effects of stimulus (deviant, standard) and group (MDD, CON), Kolmogorov-Smirnov two-sample tests were used. The Kolmogorov-Smirnov test is a nonparametric statistical test which compares the distributions of data rather than any parameter (mean, median). In MATLAB, the function *kstest2* was used for this test.

For the correlations, the Spearman’s two-tailed correlation was performed for all tests as most of the variable distributions were non-normal. The function *corr*, with type = “Spearman,” was used in MATLAB for this correlation.

To correct for multiple statistical tests, the Benjamini-Hochberg False Discovery Rate (60) was applied to all *p*-values.

## Results

### Modulation of Peak Frequency and Coefficient of Variation During the Resting State in Depression

We began our analysis with the resting state data. Over the full 3-min resting state, the mean peak frequency (PF), coefficient of variation (CV), and mean power in the theta (4–8Hz) and alpha (7–13Hz) bands was measured and statistically tested with Kolmogorov-Smirnov two-sample nonparametric tests.

**Figure 1:**
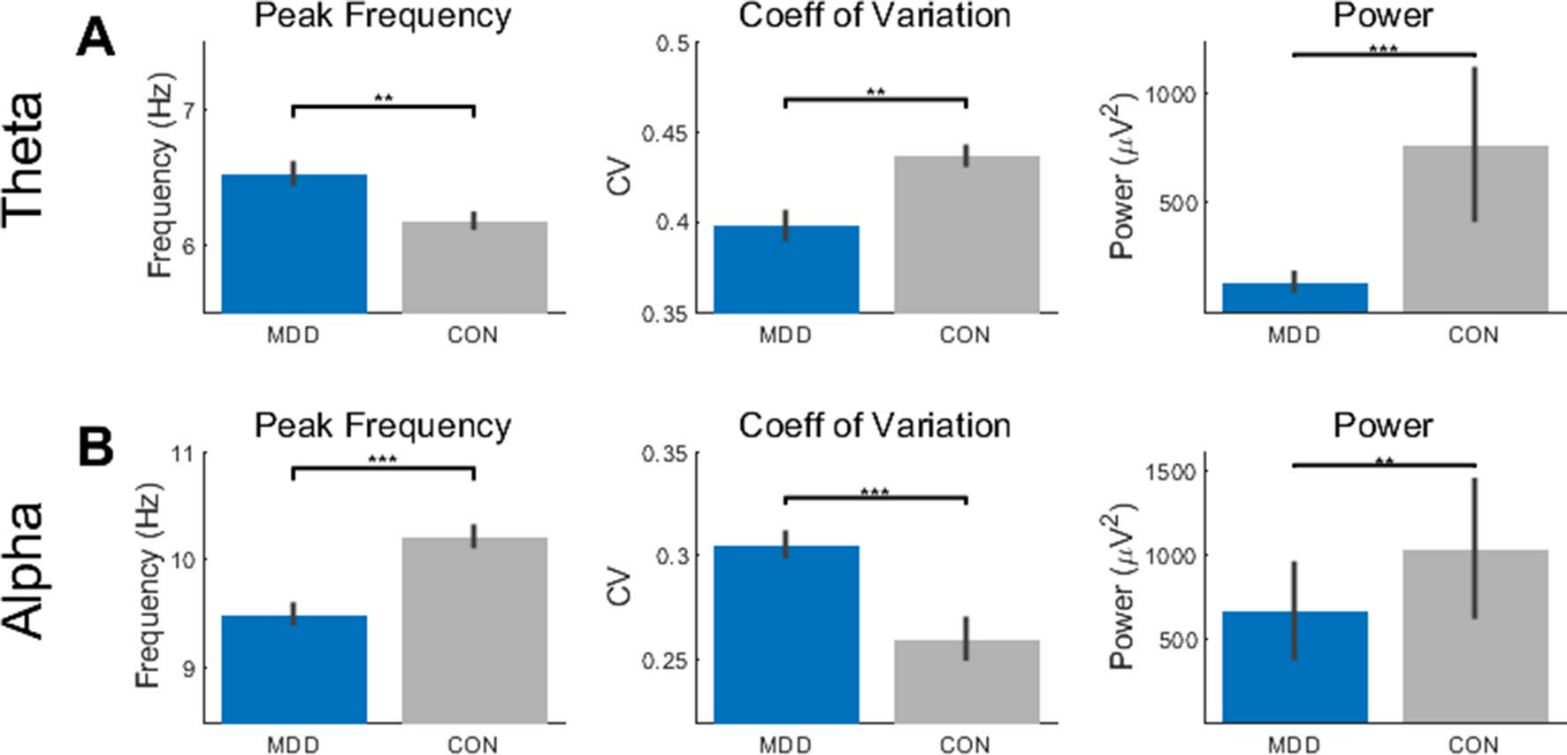
Resting state activity showed differences in theta and alpha between the MDD group and healthy controls. **(A)** The peak frequency (PF), coefficient of variation (CV), and power measures showed higher PF, lower CV, and lower power in the MDD participants than the controls. **(B)** In the alpha band, the MDD group had lower PF, higher CV, and lower power. All *p*-values are FDR corrected for multiple comparisons. **: < 0.01, ***: <0.001.

In the theta band, we first found a difference between the MDD and control participants in the PF (*D* *_28,25_* = 0.496, *p* = 0.002). We then tested to see if there was an effect of group on the CV and power and found that this was also true in the theta band (*D* *_28,25_* = 0.541, *p* = 9.021×10^−4^ for CV; *D* *_28,25_* = 0.670, *p* = 1.042×10^−5^ for power) (**Figure 1A**). In the MDD participants, the PF was higher, and CV and power lower than the healthy controls.

We then repeated the same statistical analysis for the alpha band. Again, we found a difference between MDD and controls in the PF (*D* *_28,25_* = 0.546, *p* = 3.947×10^−4^), CV (*D* *_28,25_* = 0.554, *p* = 3.947×10^−4^), and power (*D* *_28,25_* = 0.479, *p* = 0.003) (**Figure 1B**). In contrast to the theta band, the PF and power were lower and CV higher in the MDD when compared to the healthy controls.

Taken together, these data suggest opposite, or reciprocal, modulation of PF and CV in theta and alpha in MDD. High PF and low CV were found in theta while the opposite pattern — low PF and high CV — was observed in alpha. Interestingly, both alpha and theta show reduced power. Together, these findings suggest more unstable resting state activity pattern in theta and alpha in MDD.

### Trial-To-Trial Variability Reduced for Deviant Stimuli in Depression

**Figure 2:**
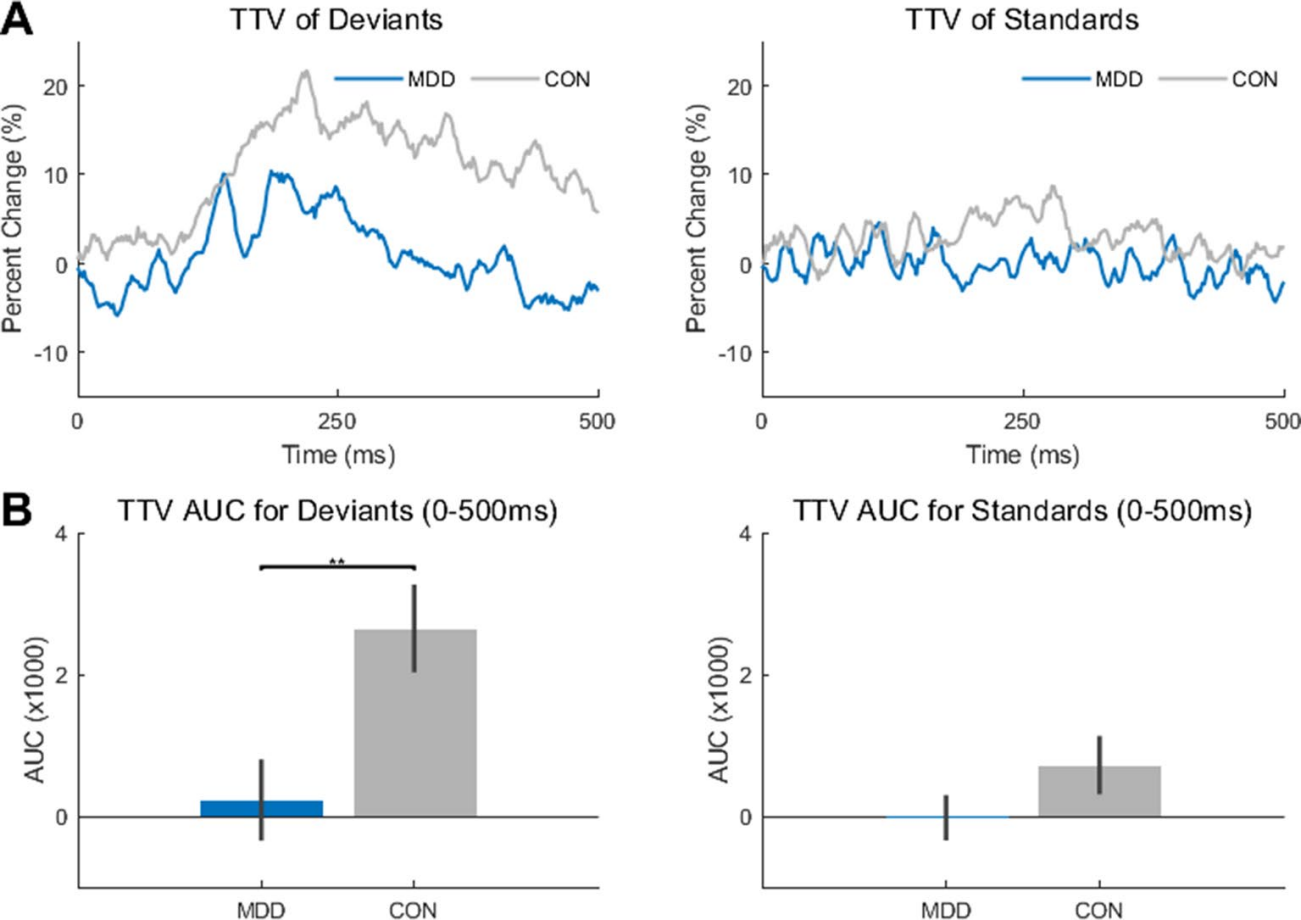
Trial-to-trial variability (TTV) showed significantly less change in variability in the MDD group for deviant stimuli. **(A)** TTV curves (80 trials) in both groups for deviant and standard stimuli. **(B)** Area under the curve (AUC) was measured between 0-500ms for both groups and stimuli. The AUC in the MDD group was significantly lower in the deviant stimuli compared to the healthy controls. All *p*-values are FDR corrected for multiple comparisons. **: < 0.01.

After completing the analysis of the resting state data, we then investigated the differences between the MDD and control group during the task. As TTV has been stated as measuring the effect of the stimulus to decrease/increase the ongoing spontaneous activity of the brain, we wanted to investigate whether there was an effect of group (MDD, control) or stimulus (deviant, standard) on TTV. To do so, the AUC of the TTV curve between stimulus onset and 500 ms at electrode Fz was calculated (**Figure 2A**).

A two-sample Kolmogorov-Smirnov test found a difference in AUC between the MDD and control participants when presented with the deviant stimuli (*D* *_26,25_* = 0.489, *p* = 0.005), with smaller AUC in the MDD participants. Therefore, there was less of a change in variability in the MDD participants when presented with the deviant stimuli than in the control participants. As the TTV was measured relative to stimulus onset, it can be inferred that the deviant stimulus made less of an impact on the ongoing spontaneous activity in the MDD group than in the control group. In contrast, no difference was found between groups in the standard stimuli (*D* *_26,25_* = 0.326, *p* = 0.105) (**Figure 2B**).

Since the deviant stimuli were only presented 10% of the time (see *Methods*), this difference may be due to the rarity of the stimulus; the control participants had more of a change in variability as they were presented with a rarer stimulus, not because they were presented with a rarer stimulus also relevant to the task. To determine if this was the case, we did the same analysis on the novel stimuli (baby cry, so environmental non-target auditory stimulus) which were also presented for 10% of the trials.

We found no difference between MDD and controls for these novel stimuli (*D* *_26,25_* = 0.169, *p* = 0.823). This result supports the idea that the difference found in deviant stimuli is not simply because they are more infrequent than the standards, as were that true, a similar difference would also exist in the novel stimuli. This was not the case.

In sum, TTV change after stimulus onset was reduced in the task-relevant deviant tone in MDD, suggesting decreased impact of this tone on stimulus-evoked activity. 

### Decreased Lempel-Ziv Complexity Change for Deviant Stimuli in Depression

The next step in the task-related analysis was to measure Lempel-Ziv Complexity (LZC). This was done to determine if there was an effect of group (MDD, control) or stimulus (deviant, standard) on the structure or pattern of the timeseries, which LZC measures. LZC (no units) was measured in 300 ms windows before and after stimulus onset. The difference was calculated by subtracting the prestim from the poststim as we wanted to see if the arrival of the stimulus coincided with a change in LZC and by what amount. It was tested, for each stimulus, for statistical difference between the two groups of participants with the two-sample Kolmogorov-Smirnov test.

**Figure 3:**
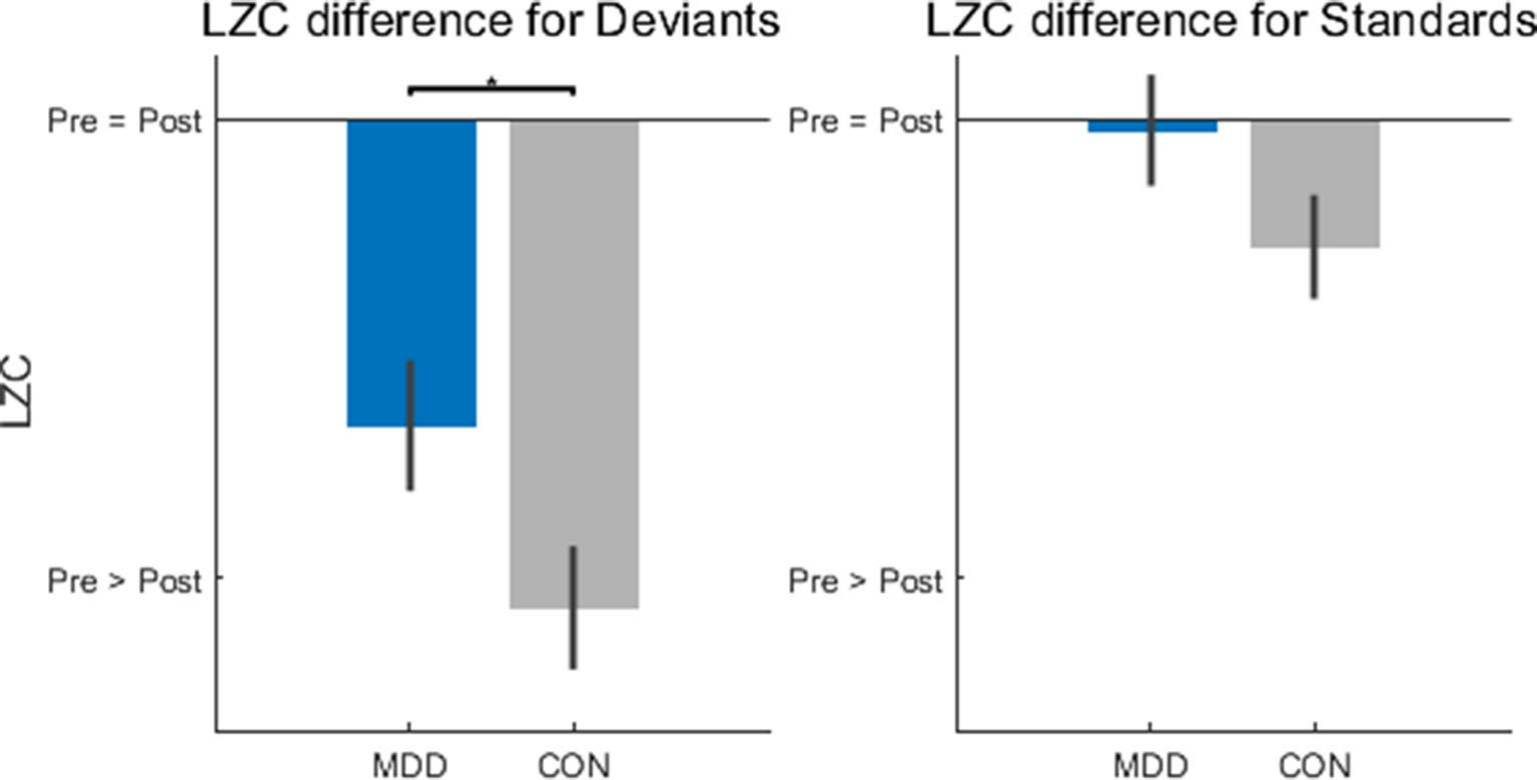
Lempel-Ziv Complexity (LZC) changes after stimulus onset were significantly lower in the MDD group for deviant stimuli. LZC was measured in 300ms time windows before and after stimulus onset. When the difference was calculated (poststim minus prestim), for deviant stimuli the MDD group showed less of a decrease when compared to healthy controls. All *p*-values are FDR corrected for multiple comparisons. *: < 0.05.

After calculating the difference—poststim minus prestim—there was found to be a difference between groups in the deviant stimuli (*D* *_26,25_* = 0.402, *p* = 0.049), but not in the standard stimuli (*D* *_26,25_* = 0.308, *p* = 0.133) (**Figure 3**). As we did in the TTV AUC above, we calculated the LZC difference in the novel stimuli to see if the difference found in the deviant stimuli was only because they are rarer than the standard stimuli. We found no difference between MDD and controls for the novel stimuli (*D* *_26,25_* = 0.183, *p* = 0.742). If there had been a difference between groups here, the difference found in the deviant stimuli would be due to the infrequent presentation of the stimulus, not due to the stimulus itself; we would conclude that the MDD group responded differently to rare stimuli, not task-related rare stimuli. As there was found to be no difference between the groups, we can infer that the finding in the deviant stimuli is not due to its frequency.

In the deviant stimuli then, the change in LZC was larger in the control participants than in the MDD group; MDD participants had less of a LZC decrease after hearing the deviant stimulus than healthy controls. One final statistical test determined that the prestim LZC for both stimuli did not differ between groups (*D* *_26,25_* = 0.182, *p* = 0.752 for deviants and *D* *_26,25_* = 0.131, *p* = 0.752 for standards). This finding is important to consider as it is the degree of change that is relevant; this indicates that the MDD subjects show decreased propensity of change when presented the deviant stimulus.

In sum, MDD patients showed less changes in LZC from the prestim period to the poststim period in deviant stimuli. This again suggests decreased impact of the external stimulus on stimulus-evoked activity which, as the LZC findings suggest, is related to decreased propensity of prestimulus activity for change by the external stimulus. 

### Frequency and Power Sliding in the Task Showed Differences in Depression

Our final task-related analysis was to measure the effect of group (MDD, control) and stimulus (deviant, standard) on change in peak frequency (PF) and power (PS) after stimulus onset. To do so in the theta and alpha bands after stimulus onset, the AUC in a 100 ms interval was calculated.

**Figure 4:**
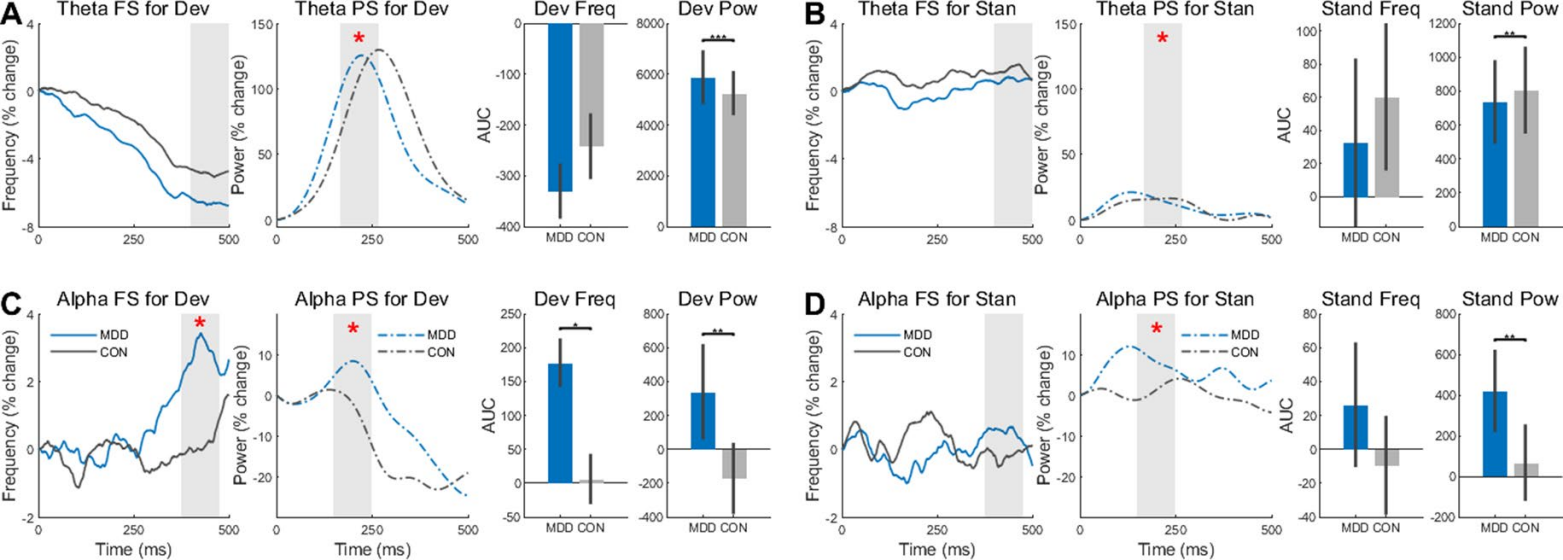
Frequency Sliding (FS) and Power Sliding (PS) found greater changes to deviant stimuli in the MDD group for theta and alpha frequency bands. **(A)** In the deviant stimuli, Theta PS was higher in the MDD group and lower **(B)** for standard stimuli. **(C)** In the alpha band, both FS and PS were significantly higher for the deviant stimuli in the MDD group. **(D)** Only the PS was higher in the MDD group for standard stimuli in the alpha band. Area under the curve for 100ms time intervals were measured. Time intervals are shown with grey shading. All *p*-values are FDR corrected for multiple comparisons. *: < 0.05, **: < 0.01, ***: <0.001.

In the alpha band, we found a difference between groups in FS when participants were presented with deviant stimuli (*D* *_26,25_* = 0.430, *p* = 0.032) (**Figure 4C**). The MDD group had larger AUC than the control group. This difference was not found when the participants were presented with standard stimuli (*D* *_26,25_* = 0.149, *p* = 0.917) (**Figure 4D**). For the PS in the same band, the difference between the MDD group and the controls was significant for both stimuli (*D* *_26,25_* = 0.460, *p* = 0.006 for deviants and *D* *_26,25_* = 0.539, *p* = 0.001 for standards), with larger AUC in the MDD participants than the controls.

Conversely, there was no difference in FS for both stimuli in the theta band (*D* *_26,25_* = 0.332, *p* = 0.188 for deviants and *D* *_26,25_* = 0*.*192, *p* = 0.685 for standards) (**Figure 4A, B**). As in the alpha band, however, there was a difference in PS in both the deviant stimuli (*D* *_26,25_* = 0.922, *p* = 2.453×10^−10^) and the standards (*D* *_26,25_* = 0.462, *p* = 0.006). The power for the MDD group was higher for the deviant stimuli and lower for the standard stimuli.

These same time intervals for AUC were analyzed again in the same way, however the absolute values of the FS—not the percent change—were measured to see if there was an absolute difference in these intervals, not just a relative difference. No difference in FS was found in the alpha band (*D* *_26,25 _*= 0.177, *p* = 0.806 for deviants and *D* *_26,25_* = 0.220, *p* = 0.806 for standards), however there was a difference in PS (*D* *_26,25_* = 1.0, *p* = 1.887×10^−12^ for deviants and *D* *_26,25_* = 1.0, *p* = 1.887×10^−12^ for standards) (**Supplementary Figures 1C, D**). The same results were found in the FS (*D* *_26,25 _*= 0.145, *p* = 0.934 for deviants and *D* *_26,25_* = 0.188, *p* = 0.934 for standards) and PS (*D* *_26,25_* = 1.0, *p* = 1.887×10^−12^ for deviants and *D* *_26,25_* = 1.0, *p* = 1.887×10^−12^ for standards) in the theta band (**Supplementary Figures 1A, B**).

In both bands and both stimuli, there was a difference in PS between the MDD and control group, while FS showed a difference between groups in percent change only in the deviant stimuli of the alpha band. Together, the increased changes, especially in PS, suggest larger instability of poststimulus activity over time in MDD than in healthy subjects. Our data on the relative difference clearly point out a reduced propensity for change by the external task-related stimulus on the ongoing spontaneous neural activity.

### Task–Task Correlation Between Trial-To-Trial Variability and Frequency/Power Sliding

After the results found above, we sought to determine if there was a relationship between the TTV and the FS/PS results. This was tested with two-tailed Spearman’s correlations.

**Figure 5:**
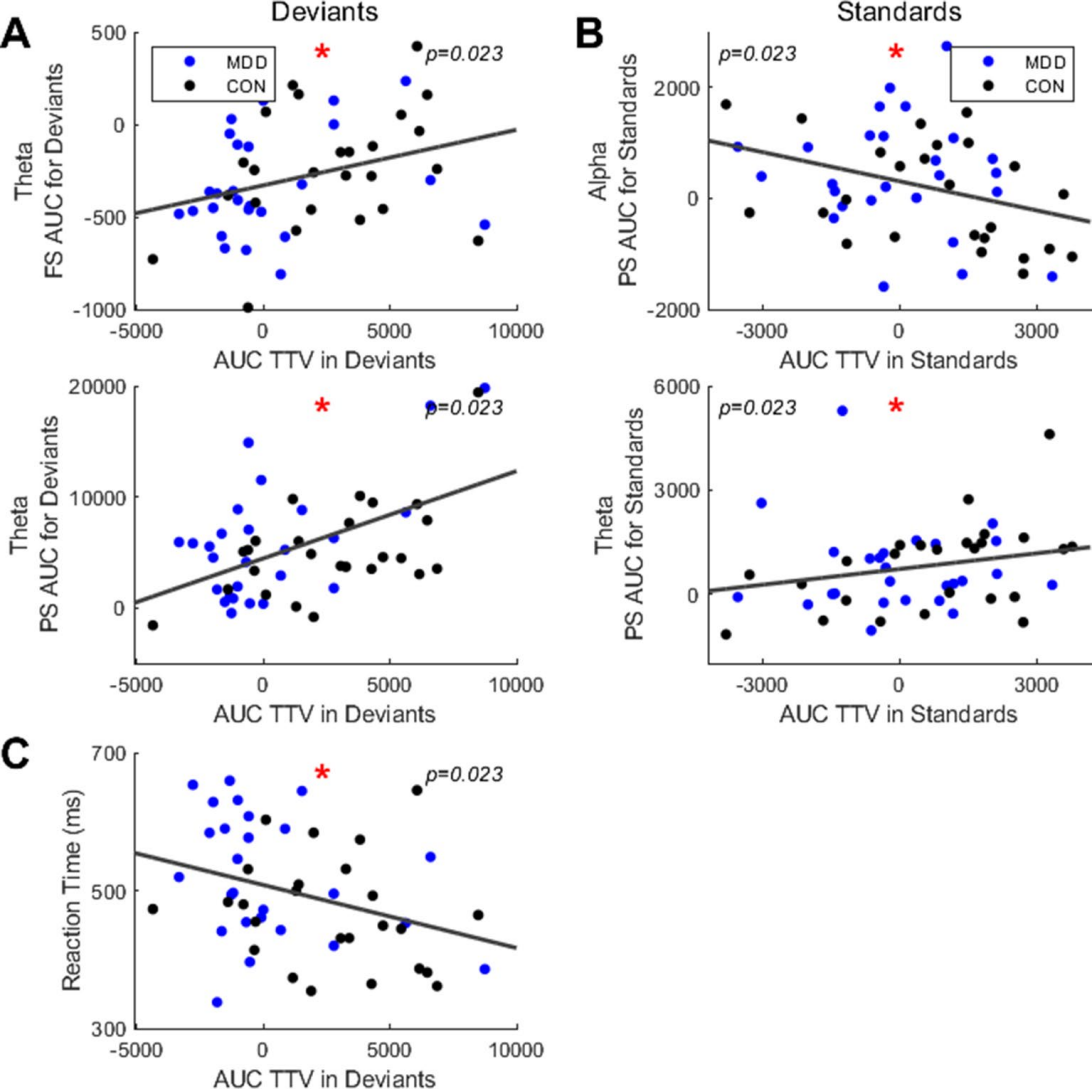
Spearman correlations found significant relationships between area under the curve (AUC) in TTV and FS, PS and reaction times. **(A)** For deviant stimuli, AUC in TTV had significant correlations across all participants with FS and PS in the theta band. **(B)** For standard stimuli, AUC in TTV had significant correlations with PS in theta and alpha. **(C)** AUC in TTV for deviant stimuli correlated significantly with the mean reaction time. All *p*-values are FDR corrected for multiple comparisons. *: < 0.05.

We first found significant correlations in the deviant stimuli between TTV AUC and both theta FS AUC (ρ = 0.334, *p* = 0.023) and PS AUC (ρ = 0.377, *p* = 0.023) (**Figure 5A**). This indicates that as the TTV variability increased after stimulus onset (larger AUC), two things happened: 1) theta peak frequency decreased; 2) theta power increased.

Next, we did the same analysis in the standard stimuli. We found a significant correlation between TTV AUC and PS AUC in the alpha (ρ = -.324, *p* = 0.023) and theta (ρ = 0.319, *p* = 0.023) band (**Figure 5B**). This indicates two points: 1) as the TTV variability increased in the alpha band, the power decreased; 2) as the TTV variability increased in the theta band, the power increased.

Finally, to link the neural activity to the behavioral data, we correlated the TTV AUC with the mean reaction times. We found a significant negative correlation in the deviant stimuli (ρ = −0.351, *p* = 0.023) (**Figure 5C**), but not in the standard stimuli. Therefore, participants with higher variability increase in the deviant stimuli had faster reaction times.

In sum, we had three findings: 1) participants had positive correlations between variability changes and theta power in both stimuli, with an increase in variability corresponding with an increase in theta power; 2) participants had a positive correlation between variability changes and PF in theta for the deviants, with an increase in variability corresponding with an increase in PF; 3) participants had a negative correlation between variability changes and PS in alpha for the standards, with an increase in variability corresponding with a decrease in PS. The TTV AUC also showed behavioral relevance with greater increases in variability showing faster reaction times.

### Rest–Task Correlations Between Resting State and Task-Related Measures

The last group of analyses we did was to test whether there was a relationship between the resting state findings and the task-related findings. To do this, two-tailed Spearman’s correlations, with the resting state measures as the independent variables, were done.

**Figure 6:**
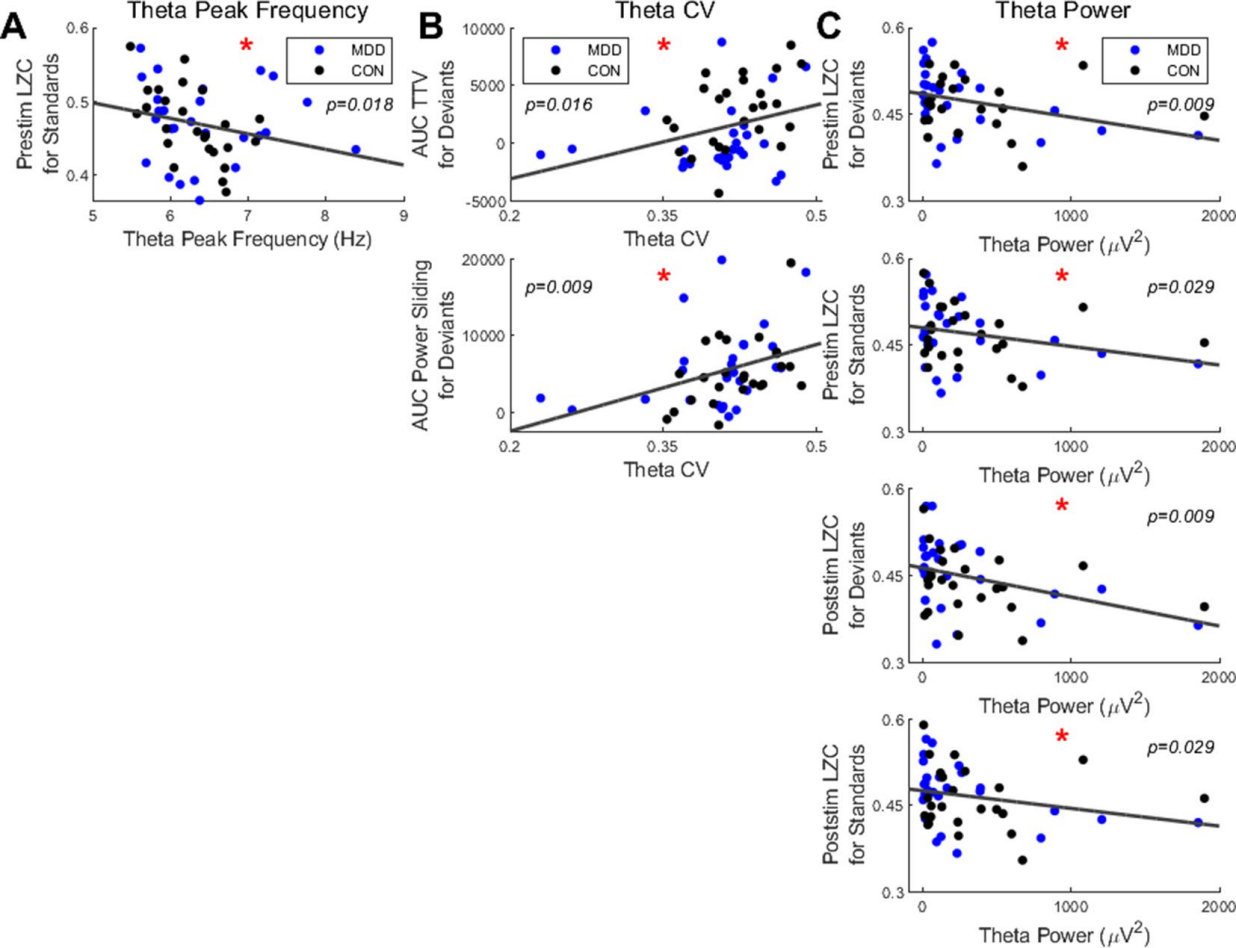
Spearman correlations found significant relationships between resting state measures in the theta band and LZC in both stimuli and PS and TTV AUC in deviant stimuli. **(A)** Resting state theta PF had a negative correlation with prestim LZC in standard stimuli. **(B)** The CV in the theta band had significant correlations with PS and TTV AUC for deviant stimuli. **(C)** Resting state theta power had significant negative correlations with both pre- and poststim LZC in both bands. All *p*-values are FDR corrected for multiple comparisons. *: < 0.05.

We first found a significant negative correlation between the resting state PF in the theta band and the prestimulus LZC for the standard stimuli (ρ = −0.345, *p* = 0.018) (**Figure 6A**); participants with higher PF had lower prestimulus complexity when presented with standard stimuli.

Next, we correlated the CV with the task-related measures. In the theta band we found a significant positive correlation between the CV and the TTV AUC in the deviant stimuli (ρ = 0.360, *p* = 0.016). A significant positive correlation between the CV and the PS for deviants was also found in the theta band (ρ = 0.420, *p* = 0.009) (**Figure 6B**). Therefore, the greater the dispersion around the mean in the theta band, as measured by the CV, the greater the a) variability increase and b) theta power increase for the deviant stimuli.

To finish, we correlated the resting state theta power with the task-related measures. We found significant negative correlations with both the pre- and poststimulus LZC in both stimuli: prestimulus LZC was ρ = −0.411, *p* = 0.009 for deviants and ρ = −0.311, *p* = 0.029 for standards; poststimulus LZC was ρ = −0.397, *p* = 0.009 for deviants and ρ = −0.308, *p* = 0.029 for standards (**Figure 6C**). Therefore, participants with higher resting state theta power had lower complexity both before and after the stimulus was presented in both stimuli.

In sum, only the measures in the resting state theta band correlated with the task measures; significant correlations were found between a) power and both pre- and poststimulus LZC in both stimuli; b) CV and both TTV AUC and PS in deviant stimuli only; c) PF and prestimulus LZC in standard stimuli only. This suggests that the increased instability of the resting state in MDD is carried over to the pre- and poststimulus activity, where it is manifest in decreased responsiveness to external stimuli as measured by decreased changes in LZC and TTV.

## Discussion

The main findings of our combined rest-task EEG study in MDD are as follows: i) opposite changes in peak frequency (PF) and coefficient of variation (CV) in theta and alpha ranges in MDD; ii) decreased neural response to deviant stimuli in MDD as measured by the change in LZC and TTV, and an increased change in FS and PS in the alpha band; iii) significantly different PS change in both stimuli and bands with theta power in standard stimuli lower in MDD and the other stimulus and band showing a higher change in MDD; iv) significant correlation of the dynamic resting state measures with stimulus-evoked dynamics.

Together, our findings show how differences in the resting state’s temporo-spatial dynamics, as seen here in participants with MDD, are carried over and thus shaping subsequent stimulus-evoked activity as has been previously shown in healthy participants (23, 24). MDD can thus be characterized by atypical temporal dynamics of rest–stimulus interaction—specifically in the theta and alpha frequency bands—during attention-demanding stimuli such as the deviant tone in our auditory oddball paradigm. 

### Resting State—Reciprocal Modulation of Theta-Alpha Dynamic

EEG resting state studies have demonstrated differences between participants with MDD and healthy controls (5, 7, 18, 67–69), among them decreased selectivity of functional connectivity during the resting state in MDD, and some with a particular emphasis on the theta and alpha frequency bands (19–20, 21, 70).

Our findings in the theta band is consistent with previous studies and symptoms of MDD. Depression has been related to neuronal atrophy in the hippocampus (71) and almost the complete absence of long-range temporal correlations in the theta band (19). Theta oscillations have been closely related to activity of the hippocampus (72), and a common symptom of depression (1) is memory impairment (73), which has been localized to the hippocampus (74, 75). These studies link theta, the hippocampus and symptoms of depression with our results which may be due to issues in the hippocampus or in the wider limbic system (67).

To our knowledge, no studies have examined peak frequency changes in MDD specifically, however a recent study found participants with first episode psychosis to have lower alpha peak frequency during the resting state (17). Other alpha findings include a study that found decreased alpha activity in participants with MDD (76). Our study replicated these findings and extend them significantly. 

Applying the measurement of PF, in conjunction with a variance measure—coefficient of variation (CV)—to resting state data, we could show opposing patterns of these two measures in theta and alpha. The CV is a measurement of the dispersion around the mean, with an increase in the CV corresponding to an increase in the dispersion of the data in the variable being measured. Also, since it divides the standard deviation of the data by the mean (Equation 1), it normalizes for individual mean values, thus negating any differences. As this measure was applied to the PF during the resting state, a lower CV indicates more stability and uniformity of the PF during the resting state. 

We did not specifically examine the inter-dependence of theta and alpha dynamics to their respective psychological functions such as memory recall (theta) (52, 77) and attention/gating (alpha) (78–81), though a reciprocal link may exist. The shift towards slower PF and higher CV in theta may indicate that the memory recall (52, 77) is dominant over attention and gating (78–81) which may be diminished by higher PF and lower CV in alpha. This notion is speculative, though consistent with clinical symptoms long studied in MDD (1). 

### Stimulus-Evoked Activity—Intrinsic and Extrinsic Components

Though EEG studies have shown similar task-related changes in participants with MDD—decreased ERP amplitude in participants with MDD when presented with a novel task-related stimulus (2, 4, 6)—and one similar resting state finding (76), our results extend these observations by showing altered dynamics in pre- (resting state activity) and poststimulus activity and apply methods new to psychiatric data.

We show first that the TTV has significantly lower change after the deviant stimulus is presented in participants with MDD. This finding implies a decreased response to the deviant stimulus of the prestimulus activity at stimulus onset as TTV change has been thought to show how a stimulus impacts the ongoing variability of activity (23, 24, 26, 29, 82), and has shown to be different than controls in participants with autism (83). This is further supported by our second finding: decreased change in LZC after stimulus onset in MDD participants when presented with deviant stimuli, though there was no difference in the prestimulus period between groups. We therefore suggest that the prestimulus activity in MDD participants is less responsive to task-related stimuli when compared to healthy controls. 

The specific role of the prestimulus’ reactivity to this task-relevant stimulus is further supported by our PS and FS findings during stimulus-evoked activity. Both FS and PS show significantly larger relative changes [no difference in the absolute values (**Supplemental Figure 1**)], especially in the alpha band, in the patient group. This suggests the dynamic instability of poststimulus activity in MDD as the basis for these changes. The differences between the groups can be observed mainly in deviant tones, but differences in power were evident in standard tones.

We now assume that such decreased responsiveness to task-relevant external stimuli, such as the deviant tone, is related to increased internal instability with higher variance across time in the poststimulus period. Our results on FS and PS support this; though we could not analyze single-trials, we could observe increased variance in both frequency and power in a time-resolved manner after stimulus onset. The decreased responsiveness to task-relevant external stimuli—seen in the decreased TTV and LZC changes—may thus be related to instability in the spontaneous activity during the stimulus period. This can be seen in the increased FS and PS; TTV changes are theorized to have the purpose of changing ongoing spontaneous activity as a way to increase the signal-to-noise ratio in favor of the stimulus (26, 27, 29, 82, 84). Further support for this is the correlation of TTV with both FS and PS in alpha and theta. This thus connects more intrinsic measures (FS, PS as measured also during the resting state)—intrinsic as being changes of the ongoing spontaneous activity which is always present—and extrinsic–extrinsic as resulting from a stimulus from the external environment—(TTV) dynamic features of stimulus-evoked activity. 

Taken together, all three findings—1) TTV and LZC, 2) FS and PS, and 3) their correlation—suggest the following: poststimulus activity in MDD may be characterized by increased neural activity instability. This in turn may decrease its ability to react to external task-relevant stimuli from the environment. Put more generally, MDD patients may suffer from an imbalance between ongoing spontaneous neural activity (internal) and components from the environment (external) in their stimulus-evoked activity, with the internal component dominating the external one. 

This imbalance seems to have a particularly strong impact on attention-demanding task-relevant stimuli (85–91). This is in accordance with the fact that we observed the main changes during the deviant tone that is more attention-demanding—participants were told to respond to it—than the standard. We therefore hypothesize that the high degree of intrinsic noise from the ongoing spontaneous activity during stimulus-evoked activity (increased FS and PS differences) may make it impossible for the depressed participants to suppress this intrinsic noise when the stimulus is presented. They are required to devote attention to, and thus process, the task-relevant external stimulus (here the deviant tone) which we see in the decreased change in TTV and LZC in the healthy participants. Maybe, MDD participants are not capable of doing so, or doing so to the degree necessary; stimulus-evoked activity may be simply too noisy for attention-demanding stimuli to be properly processed. Our aim in this study was to determine if there were differences between groups, which we did, so a future study must be done specifically to examine this signal-to-noise ratio hypothesis in participants with MDD.

### Rest–Stimulus Interaction—Resting State Dynamics Shape Intrinsic and Extrinsic Components of Stimulus-Evoked Activity

Our results, as measured by FS and PS, suggest differences in theta and alpha dynamic activity in the MDD participants during the poststimulus period. These dynamics may influence other stimulus-related activity which can be measured by TTV and LZC. The question for us, then, is what is the source of such dynamics in the ongoing spontaneous activity? One possibility is the resting state itself; its dynamics may transfer and shape that of the poststimulus period, as it has been shown that the state of this spontaneous activity when the stimulus is presented has a differential impact on the stimulus-evoked activity (23, 24).

We explored such a hypothesis by correlating resting state measures (PF, CV, and power) with those of stimulus-evoked activity (TTV, LZC, FS, and PS). As expected, this yielded significant correlations; these findings suggest a direct relationship between resting state dynamics and those of the stimulus-evoked activity as measured by LZC and TTV; the former is carried over to the latter, as was previously shown in healthy participants only (23, 24, 29). Our aim in this study was to determine if there was a difference between groups, but the strength and boundaries of this transfer from ongoing spontaneous activity to stimulus-evoked activity, as well as a possible role in psychiatric symptoms, however, must be examined specifically in a future study.

These findings suggest that the resting state dynamics in the MDD group, especially in the theta band, is related to the decreased reactivity to task-relevant external stimuli which was measured by TTV and LZC changes. This may be mediated by the resting state dynamics as measured by FS and PS. Together, these findings suggest that the differences in the resting state temporal dynamics of MDD participants are carried over to subsequent stimulus-evoked activity. 

### Physiologic–Mechanistic Basis of Atypical Rest–Task Interaction in MDD

It has been shown, through computational modelling (32) and human/animal studies (16, 92, 93) that changes in input intensity at the neural network level can be measured by changes in PF, not power, measured on the scalp using EEG. It was also shown that changes in PF — as a result of changes in input to the corresponding neural network—can produce changes in the networks’ subsequent response to inputs (16, 32, 94). This leads to sensitization/desensitization of the neuron by lowering/raising the threshold to generate an action potential (32). 

Together these findings suggested a hypothesis (16, 32): changes in PF is a gain–control mechanism. Briefly, faster oscillations—which would have higher PF—allow for accurate but restrained responding as the neurons must meet a higher threshold before an action potential is generated. At the same time, slower oscillations—lower PF—enable fast responding as the threshold to generate an action potential is lower. The drawback to this lower threshold is that these oscillations may be guided by noise as it permits a response to weaker inputs, so more inputs will meet this threshold.

In addition, input from a relatively slower oscillation will have a longer time window into which to integrate voltage; one cycle is longer in a slower frequency such as 9 Hz than a relatively faster frequency like 10 Hz. Therefore, bands with decreased PF have longer time windows in which action potentials can occur [relevant for detecting temporally coincidental inputs at the synapse (14)]. This in turn leads to greater spike timing variability (32). This adaptive mechanism (16) responds to the demands presented by a task and may adjust the sampling rate—increasing the PF would allow for more cycles in the same period of time—of incoming information required by this task. This is supported by findings from several studies (95, 96).

How does this apply to our results? Our findings of opposing differences between groups in theta and alpha show that PF is directly related to uniformity; MDD participants had higher theta PF and a lower, more stable CV, and lower alpha PF and a higher, less stable CV. The power in both bands was lower in the patient group, illustrating the nonlinear relationship between PF and power as shown previously (32, 97).

We found that the group with the faster PF also had the more stable PF, as measured by the CV. In the patients with depression, therefore, the resting state theta band was faster, more uniform—thus stable—and less powerful than the controls. In the resting state alpha band, the patients with depression were slower, less stable and less powerful, which is consistent with previous studies (76). This suggests an extreme instance of reciprocal inter-dependence in MDD between theta and alpha in opposite directions. 

**Figure 7:**
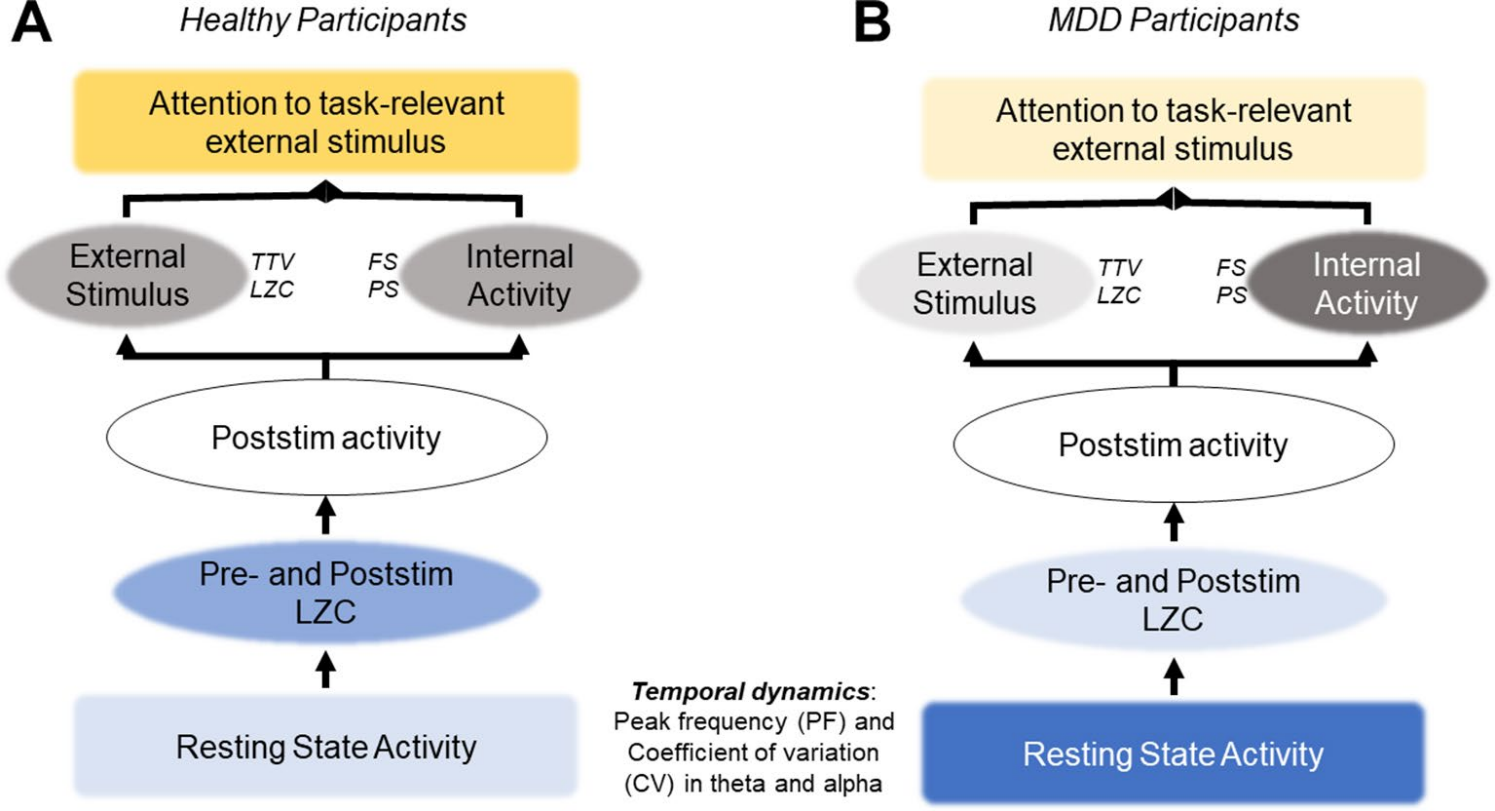
Hypothesis: atypical resting state activity leads to increased internal activity and decreased response to external stimuli. **(A)** In healthy participants, normal resting state activity allows for the quenching of ongoing variability (TTV, LZC) and an optimal signal-to-noise ratio (SNR) to respond to task-relevant stimuli. **(B)** In MDD participants, atypical resting state activity leads to higher levels of noise in the SNR, overshadowing the task-relevant stimulus. Recent studies support this hypothesis; however, it must be tested directly in future studies.

These theoretical studies that lead to the adaptive gain-control mechanism hypothesis are consistent with our results. As described above, a lower PF experiences more spike timing variability and is driven more by noise. Higher PF, in contrast, has more selective responses due to a higher threshold for generating action potentials. Our resting state results showed that the MDD group had lower PF and higher CV, but they also had a greater relative response to the deviant stimuli in the alpha band. This could be an attempt to overcome the lower baseline level and to increase the sampling rate (increase the PF), thereby increasing the information being taken in, as required by the task-relevant deviant tone. This PF increase response to the stimulus was also seen in the control group, however the baseline activity of the two groups differ (**Figure 7**), which may be the most important finding. The lower threshold in the alpha band that comes with a lower PF allows for more noise and variability generally in the signal. The signal-to-noise ratio (SNR) is then insufficient to respond to the task-relevant stimulus, despite this increase in alpha PF after stimulus onset.

This possibility may underlie the decreased changes in TTV and LZC after stimulus onset we found in the MDD group when compared with the healthy controls. It may be that the variability in the ongoing resting state alpha activity in the MDD group, specifically in this important band for sensory processing (33, 80, 98–101), due to a lower action potential threshold is too high for the PF increase seen in our results. The SNR is below what is required for changes in TTV and LZC after presentation of a task-relevant stimulus, as both these measures have been shown to relate to activity in the alpha band (29). If supported, this hypothesis would support symptoms of depression which include decreased response to sensory stimuli (1, 6), and present resting state alpha PF as a possible therapeutic target.

### Limitations

There were several limitations to this study. To begin, the relatively small number of participants in each group (<30), as well as their age (means of 54 and 46 years), made distilling out differences related to the MDD diagnosis—amid the large interindividual differences—more challenging. This was aided using the nonparametric Kolmogorov-Smirnov (KS) test which measures differences in distributions rather than the mean. This statistical test provided two advantages here: 1) it is robust to outlying participants while mean-based statistical tests are sensitive to them, and 2) it is effective with small sample sizes (less than 30 participants) (66). Therefore, though our study did have relatively small sample sizes, and there was large interindividual variability in some of our results, our use of the KS test lessened these factors.

Furthermore, a third possible factor may have accounted for the differences between groups found in our results. As noted above, the mean age difference between the MDD and control group was just under 10 years. This difference may have contributed, at least partially, to the differences we found (102–104). In fact, it may be the case that a third unknown factor contributed to our findings of differences between the MDD and control groups. Replication of this analysis would determine if this were to be the case.

Next, the recording of data would benefit in future studies from high density EEG or MEG rather than the 32 channels used here. High-density recordings would allow for source localization and analyses in source space, as well as greater topographical specificity of activity on the scalp.

Finally, though the task paradigm presented a simple sensory stimulus with behavioral response (reaction times), the short intertrial intervals may have prevented some of the measures in our analysis (FS, PS, LZC) from a return to baseline before the presentation of the subsequent stimulus. As the intertrial interval was only one second long, the stimulus-evoked activity may have carried over into the trial of the subsequent stimulus; the EEG activity measured just after onset of that next stimulus would have been the result of a) the ongoing spontaneous activity, b) activity evoked by the stimulus just presented, and c) activity carried over from the stimulus of the previous trial. For this reason, the analyses described here should be applied to other task paradigms and datasets to verify the results. Until then, these findings are preliminary.

## Conclusion

We here investigated combined rest and task EEG in major depressive disorder (MDD). Our findings show differences in resting state dynamics between the patient group and healthy controls, and that this is carried over to subsequent stimulus-evoked activity. We therefore tentatively postulate atypical temporal dynamics of rest–stimulus interaction in MDD. This difference in MDD patients may predispose them to respond in an atypical way to task-related external stimuli. 

## Data Availability Statement

The datasets generated for this study are available on request to the corresponding author.

## Ethics Statement

The studies involving human participants were reviewed and approved by University of Ottawa Institute of Mental Health Research Ethics Board. Written informed consent to participate in this study was provided by the participants themselves.

## Author Contributions

The data were acquired by SS, EL, and VK and the patients were referred to VK by PB. The data were analyzed by AW, AS, and GN and the manuscript was written by AW and GN.

## Funding

This work was supported by the EJLB-Michael Smith Foundation, the Canadian Institute of Health Research (CIHR), and the Hope of Depression Foundation (HDRF).

## Conflict of Interest

The authors declare that the research was conducted in the absence of any commercial or financial relationships that could be construed as a potential conflict of interest.

## Supplementary Material

Supplementary Figure 1 Frequency Sliding (FS) and Power Sliding (PS) between groups for both stimuli in absolute values, not relative change. **(A)** Theta for deviant stimuli. **(B)** Theta for standard stimuli. **(C)** Alpha for deviant stimuli. **(D)** Alpha for standard stimuli. *: < 0.05, **: < 0.01, ***: <0.001.
