# Supplementary figures and images for: Atypical Temporal Dynamics of Resting State Shapes Stimulus-Evoked Activity in Depression—An EEG Study on Rest–Stimulus Interaction
Source: Front Psychiatry. 2019 Oct 15;10:719. doi: 10.3389/fpsyt.2019.00719 (PMC6803442; doi:10.3389/fpsyt.2019.00719)

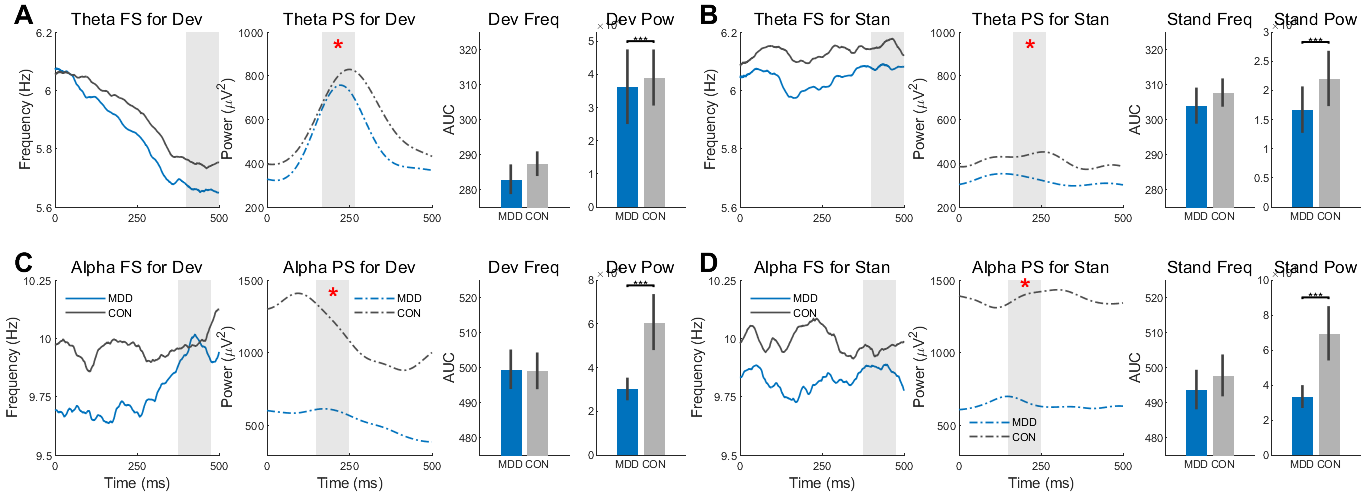

Supplement: Supplementary Figure 1 — Frequency Sliding (FS) and Power Sliding (PS) between groups for both stimuli in absolute values, not relative change. (A) Theta for deviant stimuli. (B) Theta for standard stimuli. (C) Alpha for deviant stimuli. (D) Alpha for standard stimuli. *: < 0.05, **: < 0.01, ***: <0.001. [file Image_1.tif]
